# Patient experience with sacral neuromodulation for faecal incontinence — a multi-centre, longitudinal cohort study

**DOI:** 10.1007/s00384-025-04870-5

**Published:** 2025-04-02

**Authors:** Matthew P. Irwin, Yang Yu, Catherine E. Turner, Kevin C. Ooi, Matthew J. Morgan

**Affiliations:** 1 Department of Colorectal Surgery, Bankstown-Lidcombe Hospital, Sydney, Australia; 2 School of Clinical Medicine, University of New South Wales, Sydney, Australia; 3 School of Medicine, The University of Sydney, Sydney, Australia

**Keywords:** Sacral neuromodulation, Faecal incontinence, Cohort study

## Abstract

**Purpose:**

Sacral neuromodulation (SNM) is an established treatment for faecal incontinence. This study analyses patient experience with SNM beyond quality of life and incontinence scores to better understand patient expectations and improve patient selection.

**Methods:**

Patients receiving SNM for faecal incontinence at three Australian sites from 2013 to 2023 were subject to cohort analysis. St. Mark’s Incontinence Score (SMIS) and Rapid Assessment of Faecal Incontinence Score (RAFIS) assessed incontinence and quality of life. Thematic analysis of structured interviews qualitatively assessed patient experience.

**Results:**

Seventy-one patients aged 52–86 years (*M* = 69) experienced SNM and 56 agreed to interview at median 6-year post-procedure. Forty-five (63%) proceeded to permanent SNM and progression was not influenced by age, sex, culture, insurance status or presence of anal sphincter defect. Thirty-nine (87%) retained their neuromodulator with battery life *M* = 6.5 years, 95% CI [5.2, 7.8]. Permanent SNM improved incontinence (*P* < 0.01) and quality of life (*P* < 0.01). Forty-eight (86%) patients desired 30-min education pre- and post-procedure. Thirty-seven (86%) desired follow-ups at 1 month, 12 months and at battery depletion. Twenty (36%) had initial reservations which resolved in all but one patient. Twenty-one (54%) remained dependent on others for neuromodulator customisation and this dependence increased with age (*P* = 0.02). Fifty (89%) recommend SNM to others, despite 8 (14%) regretting their personal experience.

**Conclusion:**

SNM continues to improve faecal incontinence and quality of life. Initial reservations usually resolve and most patients recommend it to others. While adequate patient education and follow-up is not onerous to achieve, most patients remain dependent for neuromodulator customisation.

**Supplementary Information:**

The online version contains supplementary material available at 10.1007/s00384-025-04870-5.

## Introduction

Faecal incontinence (FI) is the unintentional passage of bowel content and can have a devastating impact on daily life [1]. It has a global prevalence of 8% with a predominance amongst females and individuals aged > 60 years [2]. Initial treatments aim to improve stool consistency and coordination of defaecatory muscles through dietary changes, bulking agents, anti-motility agents, enemas, and biofeedback. For persistent symptoms, sacral neuromodulation (SNM) is an established treatment with well reported long term outcomes [3].

SNM involves a minimally invasive procedure to place electrodes through S3-4 foramina to stimulate the nerve roots. The procedure has low morbidity and is typically performed in two stages [4]. The first stage involves temporary electrode placement to test therapeutic effectiveness. The second stage involves permanent lead placement then connected to a neuromodulator implanted into subcutaneous fat in the superior gluteal region. While the mechanism by which SNM achieves improved continence is largely unknown, the result is increased sphincter tone, rectal capacitance and coordination of pelvic floor muscles [5].

Previous studies have established the benefit of SNM on incontinence and quality of life scores [6–14]. This study is the first to analyse the patient experience beyond these scores to facilitate a better understanding of patient expectations and improve patient selection.

## Methods

**Fig. 1:**
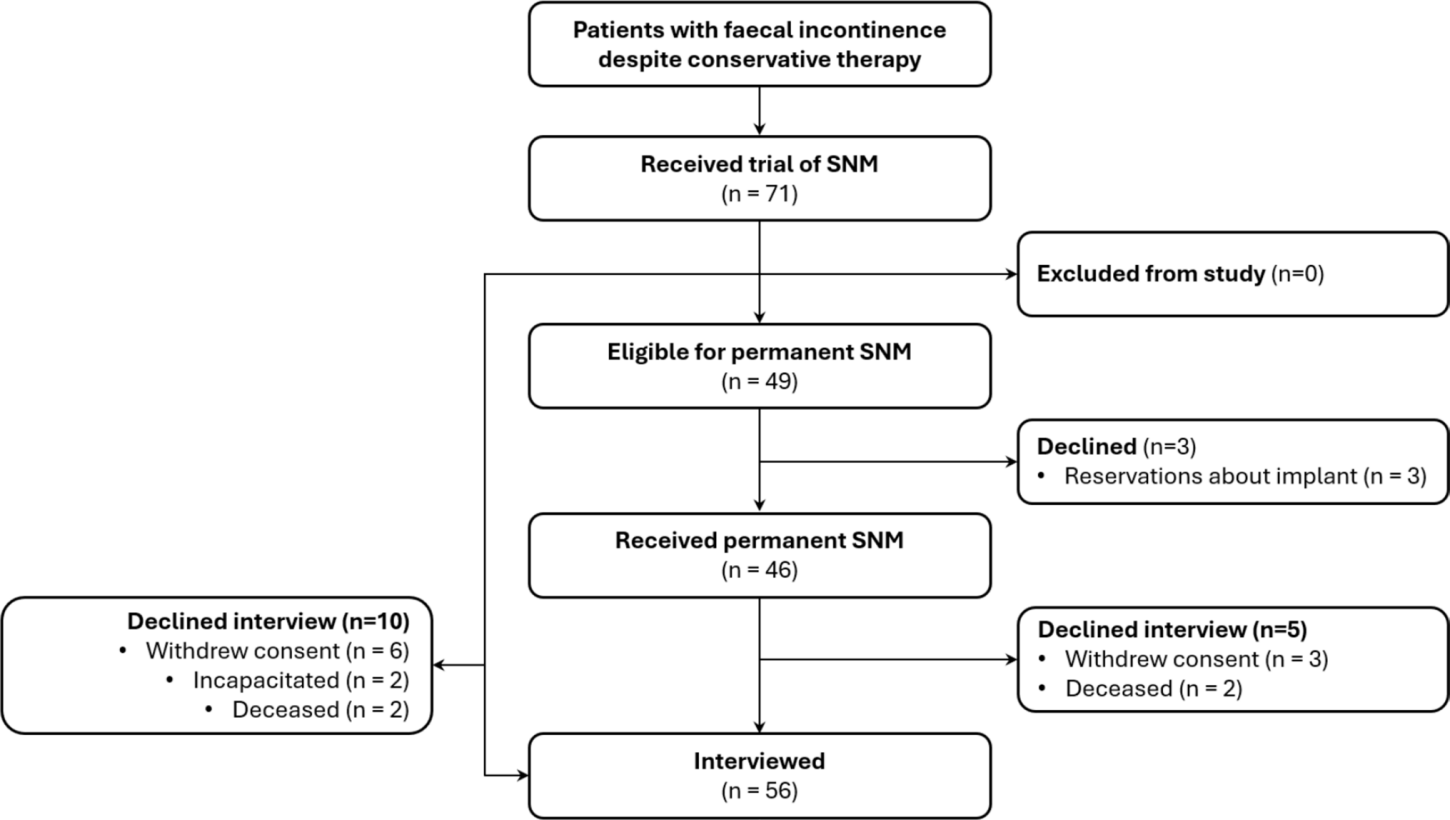
STROBE flow chart*. STROBE, *strengthening the reporting of observational studies in epidemiology

Patients over 18 years of age with FI for > 3 months despite dietary changes, bulking agents and biofeedback were invited to participate in a prospective cohort study when booked for trial of SNM at a tertiary referral hospital and two private hospitals in Sydney, Australia, between 2013 and 2023 (Fig. 1). There were no differences in selection criteria between public and private hospitals.

Baseline incontinence and quality of life (QOL) was assessed with St. Mark’s Incontinence Score (SMIS) and Rapid Assessment of Faecal Incontinence Score (RAFIS). In addition to the standard consultation, patients assessed as appropriate for SNM received 30-min of education from a combination of their surgeon and a neuromodulator device representative regarding faecal incontinence, SNM, and after care. This session was delivered face-to-face and utilised a model pelvis to explain the anatomy, a short animation to explain normal and abnormal defaecation, and a demonstration neuromodulator to explain insertion and post-operative customisation. The surgeon assumed responsibility for session content, and the device representative assumed responsibility for equipment provision and a demonstration of post-operative customisation. Patients were then required to complete a bowel diary documenting the time and consistency of each stool, plus the presence of urge or incontinence, and use of pads or medications in the two weeks prior to temporary electrode placement. Patients with an incomplete bowel diary had their procedure postponed until adequately completed.

A senior colorectal surgeon (CET) placed temporary electrodes into bilateral S3-4 foramina under sedation with the patient in prone position. Placement was standardised using surface anatomy, intraoperative image intensifier, and elicitation of motor reflexes including the bellows response and plantar flexion of the hallux [15]. Temporary electrodes were connected to an external neuromodulator which was checked by the device representative and the patient educated about its use. Patients were instructed to maintain their modified diet and continue bulking agents ± anti-motility agents during the trial.

Patients continued their bowel diary until surgeon review two weeks following the procedure at which temporary electrodes were removed and patients offered permanent electrode placement in the event of a 50% reduction in each of incontinence episodes, use of pads and anti-motility agents. Permanent electrode placement was performed by CET in the same manner as temporary electrode placement except for them being connected to a Medtronic Interstim II (Minneapolis, USA) neuromodulator implanted into subcutaneous fat in the superior gluteal region. The neuromodulator was then paired with a feature-limited mobile device and checked by the same device representative who further educated the patient about its use. The above neuromodulator was used as it was the only device approved by the Therapeutics Goods Administration for the entire study period. Patients were instructed to maintain their modified diet and continue bulking agents and pelvic floor exercises [16].

Patients had routine surgeon review at 1 month, and every 6 months until battery depletion during which complications were recorded and incontinence and quality of life scores were reassessed. The same device representative provided ongoing technical support for patients.

Semi-structured interviews assessing the patient experience from time of referral for SNM to neuromodulator battery depletion were conducted by author MPI according to the COREQ-32 framework (Appendix 1). A guide of 18 questions was informed by a pilot study and used to elicit adequacy of education, training and follow-up; reservations around implantation; degree of independence with neuromodulator customisation, and emotional aspects of the experience. Interviews were transcribed, de-identified, and subjected to independent inductive coding and reflexive analysis by authors, YY and MPI, with differences resolved by discussion. Researcher reflexivity was maintained by extensive field notes and returning transcripts to participants for approval.

### Statistical analysis

Patient age, sex, cultural background, insurance status, anal sphincter defect, history of obstetric trauma and abdominopelvic surgery, comorbidities, timing and location of procedures, incontinence and quality of life scores were analysed using IBM SPSS V28.0. Patients self-reported cultural background and understood it to mean what culture they most identified with. Paired *t*-test and multivariate analysis was used for quantitative data. Mid-*P*-values were reported, and significance was determined at *α* = 0.05. χ2 Goodness of Fit test was used for categorical data. Bonferroni correction was used to control for multiple comparisons.

## Results

Seventy-one primarily female (90%) patients aged 52 to 86 years (*M* = 69) experienced SNM from 2013 to 2023. Forty-six (63%) patients proceeded to permanent SNM. Three patients declined an offer of permanent SNM despite experiencing a successful trial of SNM, citing reservations about neuromodulator implantation. There was no difference in age, sex, cultural background, insurance status or history of anal sphincter defect between the trial only and permanent neuromodulation groups (*P* > 0.05) (Table 1). The mean battery life was 6.5 years, 95% CI [5.2,7.8].

**Table 1:** Patient characteristics

	Trial SNM only	Permanent SNM	
	(*n*=25)	(*n*=46)	*p*-value
Age (years)			0.83
Mean (Min, Max)	69 (52, 85)	69 (52, 86)	
Gender			0.70
Female	23 (92%)	41 (89%)	
Male	2 (8%)	5 (11%)	
Cultural background			0.79
Australian	16 (64%)	28 (61%)	
Other	9 (36%)	18 (39%)	
Sphincter defect			0.86
Present	7 (28%)	12(26%)	
Absent	18 (72%)	34 (74%)	
Repaired sphincter defect			0.25
Yes	7 (100%)	10 (83%)	
No	0 (0%)	2 (17%)	
Insurance status			0.79
Public insurance (Medicare)	9 (36%)	18 (39%)	
Private insurance	16 (64%)	28 (61%)	

### Incontinence

**Fig. 2:**
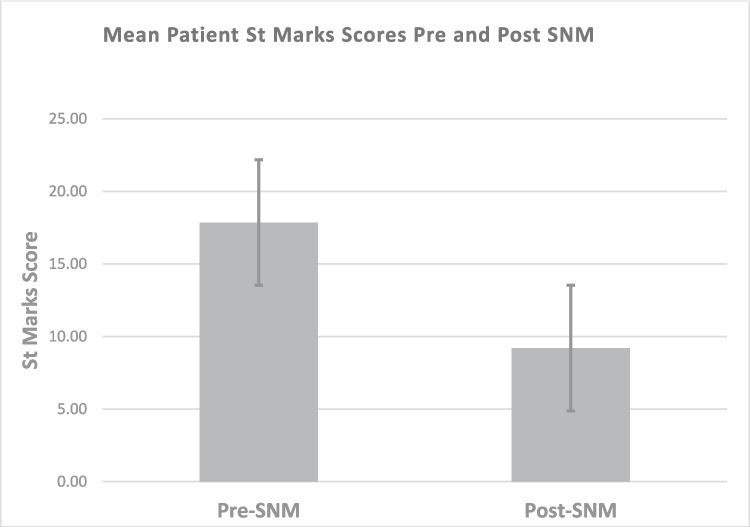
St.Marks Incontinence (SMIC) scores pre- and post-permanent SNM. Mean SMIC prior was 17.85 95% CI [18.97, 16.72] compared to post permanent SNM SMIC of 9.2 95% CI [11.10, 7.29]

Permanent SNM improved incontinence (*P* < 0.001) with an average reduction in SMIS of 9 points, 95% [7, 8] (Fig. 2). This improvement was also observed using RAFIS, where there was a difference of 5 points, 95% CI [4, 6].

### Quality of life

**Fig. 3:**
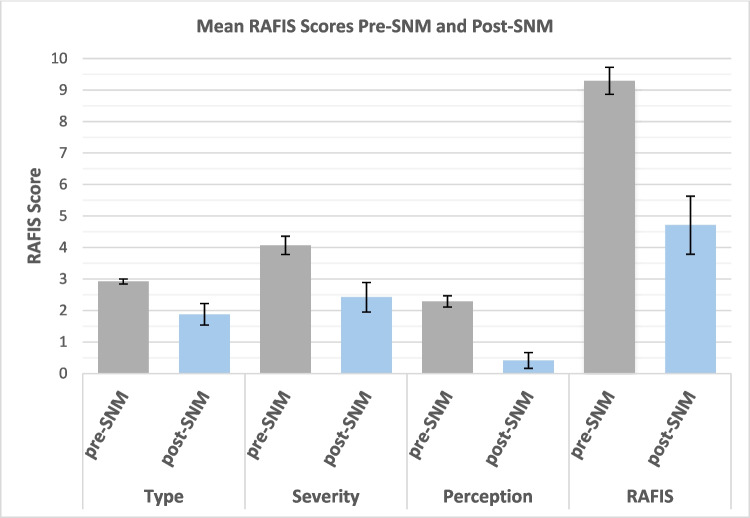
RAFIS Scores pre- and post-permanent SNM**.** Mean Pre-SNM: Type = 3.01, 95% CI [3.01,2.84], Severity = 4.07 95% CI [4.37,3.78], Perception = 2.29 95% CI [2.47,2.12], Total RAFIS = 9.29 95% CI [9.72,8.86]. Mean Post-SNM: Type = 1.87 95% CI [2.23,1.53], Severity = 2.41 95% CI [2.89,1.94], Perception = 0.41 95% CI [0.67, 0.16], Total RAFIS = 4.70 95% CI [5.63,3.78]

Quality of life improved (*P* < 0.01) with the greatest relative change in perception (− 82%) followed by severity (− 40%) on RAFIS (Fig. 3). The change in perception translated to most patients ‘feeling good’ as opposed to ‘terrible’, and the change in severity translated to most patients experiencing incontinence ‘several times a month’ as opposed to ‘several times a week’.

### Complications

Thirty-nine (87%) of 45 patients retained their implanted neuromodulator despite 20 (51%) experiencing a complication. The most common complication was pain (10, 50%) followed by haematoma (5, 25%) and infection (3, 15%). Of those with pain, 6 patients (60%) required neuromodulator repositioning and 1 (10%) required neuromodulator explantation. Half of these patients had pre-existing pain issues. Of those with haematoma, 3 (60%) required drainage. No patient with infection required explantation.

Symptom recurrence was the primary reason provided for four (67%) of the six patients who had neuromodulator explantation during the study period. The remaining two provided pain and resolution of symptoms despite a depleted battery as reasons for explantation.

### Patient experience

**Fig. 4:**
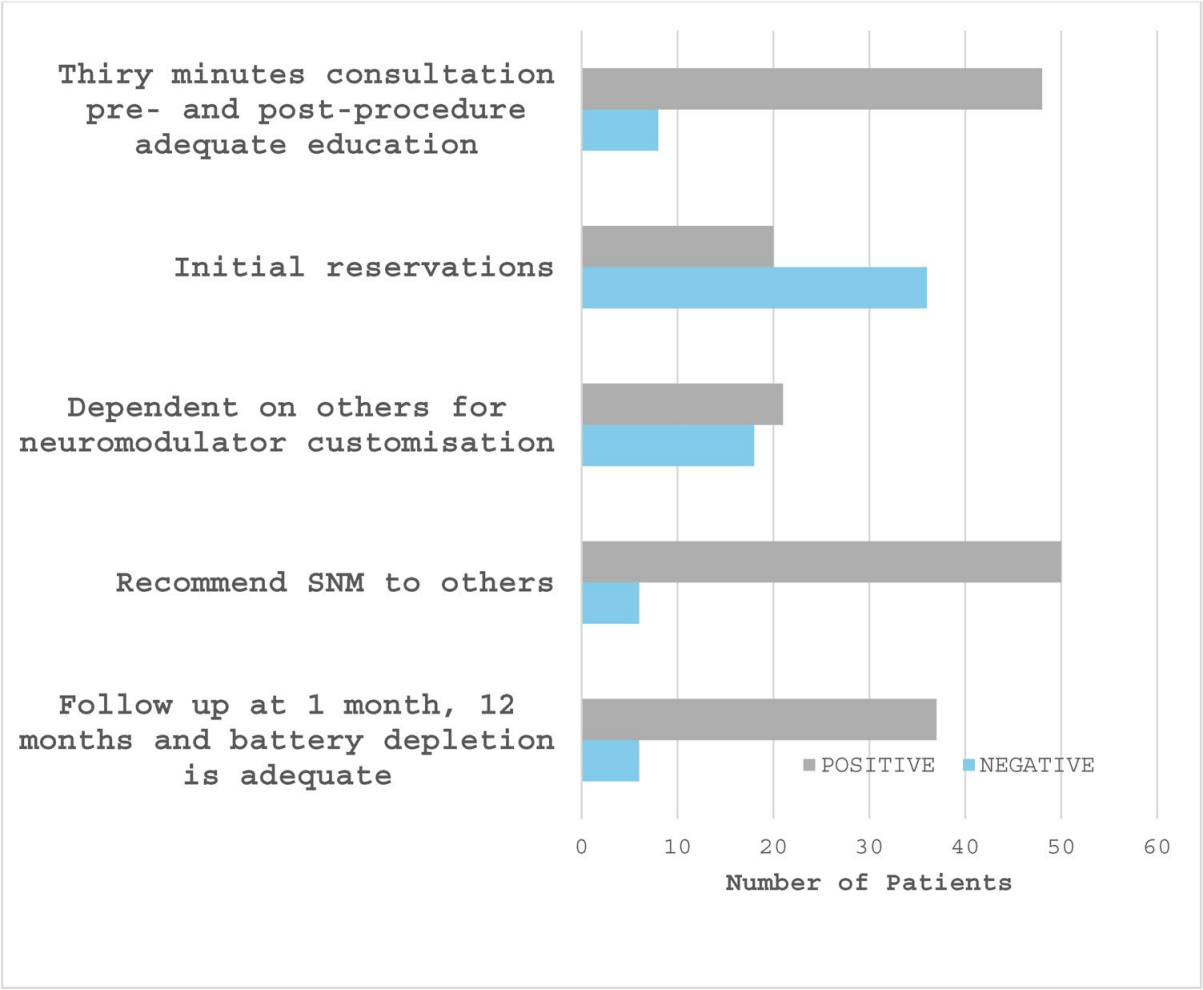
Pertinent findings from structured interviews of patients experiencing sacral neuromodulation

Fifty-six patients experiencing SNM agreed to interview (Fig. 4) at median 6 years post procedure. Concepts, themes and aggregates from these interviews are in Table 2.

**Table 2:** Data structure

	Concepts	Themes	Aggregate
Education	• Sessions improved understanding • Sessions provided hope where previous treatments had not • Duration of sessions before and after procedure was necessary and sufficient • Sessions facilitated introspection • Sessions established confidence with neuromodulator customisation	• Empowerment was achieved with education • Increased knowledge improved patient experience • Device representative was essential for managing technical minutia	• Thirty minutes education before and after procedure is desired
Reservations	• Complication from electrode insertion • Developing dependence on SNM • Discomfort from neuromodulator • Ideological opposition to implant • Vulnerability to technical malfunction • Physical and social safety with air travel • Negative anecdotes in media or on internet	• Fear of malfunction further decreasing quality of life • Fear of complications adding to social anxiety • High sensitivity of condition increases threshold to trust	• Initial reservations are common but most resolve over time
Device	• Inaccessibility of device representative • Burden on family • Fear of abandonment and helplessness • Highly specialised treatment limited options for assistance • Reluctance to learn	• Distrust of self to manage device • Reluctance to learn	• Dependence on others for neuromodulator customisation is common
Follow-up	• Comparing incontinence scores affects perception of treatment outcome • Diagnosing a depleted battery at follow-up questioned ongoing necessity of device • Follow-up provided avenue for further troubleshooting • Confidence with neuromodulator customisation maintained with follow-up • Provided follow-up schedule was adequate	• Follow-up is important to patients in them determining their treatment outcome • Treatment outcome can improve with adequate follow-up	• Follow-up at 1 month, 12 months and at battery depletion is desired
Perception	• Neuromodulator complications replaced one problem with another • Did not appreciate restriction on future imaging modalities • Desperation clouded decision making • Empathising with others is primary consideration in recommending SNM • Regret with symptom recurrence	• Empathy and desperation dictate recommendation to others	• Most patients recommend SNM, despite some regretting their personal experience

Forty-eight (86%) patients desired 30-min education pre- and post-procedure. Twenty (36%) patients had initial reservations which resolved in all but one patient who declined permanent SNM. Twenty-one (54%) patients with an implanted neuromodulator remained dependent on others for customisation which increased age predicted (*P* = 0.02) but inadequate education did not (*P* > 0.05). Fifty (89%) patients recommend SNM to others, despite 8 (14%) patients regretting their personal experience. Thirty-seven (86%) patients who received permanent SNM desired follow-up at 1 month, 12 months and at battery depletion. Patients who desired more follow-up than they received were likely to be dependent on others for neuromodulator customisation (*P* = 0.05).

## Discussion and conclusions

This study was designed to extend the evaluation of SNM for FI beyond traditional measures of incontinence and quality of life by focusing on the broader patient experience. Our primary objective was to understand patient expectations and optimise patient selection through structured interviews. From these, we have elicited that a focused 30‐min education session delivered both pre- and post-procedure is not only desired but may alleviate initial reservations and establish a foundation of confidence in participants managing their neuromodulation devices. In addition, there is a clear preference for follow-up at 1 month, 12 months and at battery depletion, underscoring the importance of continuous support in achieving sustained treatment satisfaction.

Beyond the symptomatic improvements, our findings highlight critical opportunities for enhancing patient autonomy. Although SNM significantly improved incontinence and quality of life, a notable proportion of patients, particularly older individuals, remained dependent on others for device customisation. This dependency suggests that refining patient selection criteria, developing tailored educational interventions, arranging more frequent preventative follow-up and simplifying the interface may be necessary to empower older patients to achieve independence and improve overall care. While our study’s results align with existing literature on SNM efficacy [7–9, 17, 18], the primary contribution here lies in demonstrating that targeted education and systematic follow-up can resolve initial reservations and support long-term patient engagement with therapy.

Patient characteristics were analysed in the hope of identifying predictors of SNM efficacy. However, despite a decade of recruitment, our sample size was inadequate for a robust multivariate analysis of age, sex, body mass index, cultural background, insurance status, anal sphincter defects and previous surgery. A larger cohort study by Feldkamp et al. (2021) identified low BMI as a positive predictor of efficacy [6], while another noted that male sex and previous surgery may negatively impact outcomes when success is defined as not requiring explantation [19]. Future research should aim to incorporate a larger patient population to further clarify these predictors for FI specifically.

Progression from temporary to permanent SNM (63%) was lower than reported in other cohorts [10, 17], likely reflecting our stricter bowel diary criteria and older patient population. However, the improvement in SMIS following permanent SNM was consistent with previous findings [7, 18], despite sampling patients beyond 5 years where other studies have demonstrated a gradual decline in efficacy [7, 9]. Similar improvements were observed using the incontinence component of RAFIS which we used to better discriminate quality of life than the St. Mark’s and Wexner scores without resorting to the FIQL score which presents an unacceptable English language bias for our multicultural cohort [20, 21]. By demonstrating similar efficacy in our cohort, we have supported the external validity of their experiences with SNM.

There exists a discrepancy in battery life between this cohort and initial claims from the device manufacturer. Mean battery life was found to be 6.4 years, compared to an initial manufacturer claim of 10 years. Similarly reduced battery life, with an average of 5.9 to 6 years [11, 12] was found in other studies using InterStim ™ II (Medtronic ™) for incontinence. A possible explanation for shorter battery life could be increasing neuromodulation intensity with time suggesting tolerance, lead migration or foraminal fibrosis. A shorter battery life was found to be associated with increase in hospital costs over time as patients require surgical revision to replace the battery [11]. While this is less likely to be an issue with next generation devices that can be wirelessly recharged, it significantly shortened the duration of treatment for patients in this cohort as 18% opted for removal over replacement once the battery was depleted.

Strengths of our study include the inherent benefits of a prospective cohort design, which allowed us to gather detailed and forward-looking insights. Conducted in South-West Sydney, the study reflects the region’s rich multicultural demographic, enhancing the generalisability of our findings. Our robust approach minimised bias through consistent procedural techniques, uniform use of equipment and devices and standardised patient education and follow-up by the same clinician, nursing staff and device representative. The structured interview process — with a sole interviewer, standardised questionnaires (SMIS, RAFIS) and validated responses — further ensured data reliability. Additionally, we maintained the integrity of our study by declining industry sponsorship and by having the tertiary referral hospital fund SNM for uninsured patients, thereby eliminating potential selection biases.

Limitations of the study include its modest sample size, with 71 patients recruited over 10 years and 45 (63%) progressing to permanent SNM. This reflects the reality that only severe cases of FI failing biofeedback are referred for SNM and underscores the challenges of diagnosing FI and utilising SNM as a treatment option [22, 23]. Economic factors in our local health district, where incomes are on average 23% lower than the state average, along with stigma and limited awareness about SNM, contribute to the observed referral patterns [24]. In a comparable district, only 1 in 10 FI sufferers report their symptoms to primary care, leading to under-referral to colorectal services [25]. Given their prevalence of 10%, we would expect a substantially larger number of patients to have received at least a trial of SNM, highlighting an opportunity for improved case identification and management [25].

In conclusion, this study demonstrates that SNM can be effectively evaluated beyond traditional incontinence and quality of life measures by focusing on the broader patient experience. A structured 30-min education session delivered both pre- and post-procedure, and scheduled follow-up at 1 month, 12 months and battery depletion were shown to strengthen patients’ confidence and long-term engagement with SNM. While symptomatic improvements were evident, older patients often required additional support with device customisation, indicating a need for refined patient selection criteria, tailored educational approaches and simplified interfaces. Overall, these findings reinforce the efficacy of SNM while highlighting the importance of targeted education and systematic follow-up in reducing initial reservations and promoting greater patient autonomy.

## Supplementary Information

Below is the link to the electronic supplementary material.

ESM1 (DOCX 23.2 KB)

## Data Availability

The data that supports the findings of this study are not openly available due to reasons of sensitivity and are available from the corresponding author upon reasonable request. Data is located in controlled access data storage at NSW Health.
