# Supplementary material for: Patient experience with sacral neuromodulation for faecal incontinence — a multi-centre, longitudinal cohort study
Source: Int J Colorectal Dis. 2025 Apr 2;40(1):84. doi: 10.1007/s00384-025-04870-5 (PMC11965221; doi:10.1007/s00384-025-04870-5)
Supplement: Supplementary file 1 — (DOCX 23.2 KB) [file 384_2025_4870_MOESM1_ESM.docx]

### **Appendix 1 – Consolidated criteria for reporting qualitative studies (COREQ): 32-item checklist**

**Domain 1: Research team and reflexivity**

1. Interview

MPI conducted the interviews.

1. Research credentials

MPI – BMed MD FRACS

YY – BCom BA BSc MD

CET – MBBS MS FRACS

KCO – BSc MBBS FRACS

MJM – MBBS MS FRACS

1. Occupation

MPI – General Surgeon and Colorectal Superintendent

YY – Colorectal Resident

CET – General and Colorectal Surgeon

KCO – General and Colorectal Surgeon

MJM – General and Colorectal Surgeon

1. JB – Gender

MPI – Male

YY – Female

CET – Female

KCO – Male

MJM - Male

1. Experience and training

MPI – Experienced clinician and researcher with research qualifications

YY – Qualitative researcher with research qualifications

CET – Senior clinician and researcher supervisor with research qualifications

KCO – Senior clinician and researcher supervisor with research qualifications

MJM – Senior clinician and research supervisor with research qualifications

Relationship with participants

1. Relationship established

Senior clinicians, CET, KCO and MJM, informed patients of the study and obtained consent. Participants were introduced to MPI by letter, scheduled for interview by SMS, and phoned for interview. Rapport was established via the exchange of pleasantries, gratitude for participating in the study, and a light conversation prior to interview commencement. Relationships were formed through the interview process and contact was maintained only to verify transcript accuracy. Clinician follow up was offered to participants reporting ongoing symptoms.

1. Participant knowledge of the interviewer

Participants were aware that MPI was a surgeon affiliated with the hospital that provided their treatment and that the study would ask questions about their incontinence and experience with sacral neuromodulation. Participants were encouraged to be honest as their responses were de-identified and were aware that study outcomes may be disseminated. This was explained in the participant information sheet, consent form, and at commencement of interview.

1. Interviewer characteristics

As Colorectal Superintendent, MPI wanted to determine the efficacy of sacral neuromodulation for faecal incontinence in the study population and identify predictors of success or failure to better improve patient selection and experience. This goal was communicated to, and resonated with, study participants.

**Domain 2: Study design**

Theoretical framework

1. Methodological orientation and Theory

Voluntary participation and thematic analysis of semi-structured interviews performed using Braun and Clarke's methodology.

Participant selection

1. Sampling

Purposive sampling of patients with experience of sacral neuromodulation for faecal incontinence in southwestern Sydney, Australia.

1. Method of approach

Participants approached by letter with participant information sheet and consent form, then one SMS reminder.

1. Sample size

Fifty-six participants (79%) agreed to be interviewed.

1. Non-participation

Fifteen participants (21%) declined to be interviewed. Reasons were since deceased (6) or symptom recurrence with progression to colostomy (3). Six patients remained uncontactable by letter, reminder SMS, and welfare call.

Setting

1. Setting of data collection

Interviews were conducted by phone.

1. Presence of non-participants

Only participant and interviewer +/- interpreter were present.

1. Description of sample

Individual interviews of 71 participants with experience of sacral neuromodulation for faecal incontinence from 2013-2023 in southwestern Sydney, Australia.

Data collection

1. Interview guide

A pilot interview schedule was tested on three patients suggested by CET as having had a negative, neutral and positive experience with sacral neuromodulation. From this, a semi-structured interview schedule of 18 questions was developed which elicited two incontinence scores; the adequacy of education, training and follow up; reservations around implantation; degree of independence with neuromodulator customisation, and emotional aspects of the experience.

1. Repeat interviews

There were no repeat interviews.

1. Audiovisual recording

Interviews were not recorded to encourage honest responses on this sensitive topic.

1. Field notes

Extensive notes, including quotations, were made during and following the interview to direct later reflexive analysis.

1. Duration

Interview duration ranged from 20 to 70 minutes.

1. Data saturation

The number of interviews required to reach data saturation was not able to be pre-defined given the scarcity of research on patient experience with sacral neuromodulation. Few new themes emerged from interviews 30 to 56.

1. Transcripts returned

Field notes were returned to the participant at interview conclusion for comment or clarification with few corrections required.

**Domain 3: Analysis and findings**

Data analysis

1. Number of data coders

Data was coded independently by MPI and YY.

1. Description of the coding tree

Inductive coding.

1. Derivation of themes

Reflexive analysis.

1. Software

Microsoft Word and Microsoft Excel

1. Participant checking

Despite being offered, no participants requested the findings be sent to them.

Reporting

1. Quotations presented

Examples of participant quotations were included to demonstrate findings.

1. Data and findings consistent

There was consistency between data and findings, with examples provided to assist the reader in assessing this.

1. Clarity of major themes

Major themes summarise minor themes.

1. Clarity of minor themes

Minor themes are derived from broadly positive or negative participant experiences. Within each group is significant homogeneity in experiences.

Reference: Tong A, Sainsbury P, Craig J. Consolidated criteria for reporting qualitative research (COREQ): a 32-item checklist for interviews and focus groups. Int J Qual Health Care 2007; 19, 349–357. doi: 10.1093/intqhc/mzm042
